# How Does Internal and External CSR Affect Employees’ Work Engagement? Exploring Multiple Mediation Mechanisms and Boundary Conditions

**DOI:** 10.3390/ijerph16142476

**Published:** 2019-07-11

**Authors:** Yu Jia, Jinglu Yan, Tianyuan Liu, Jun Huang

**Affiliations:** 1 School of Economics and Management, Wuhan University, 299 Bayi Road, Wuhan 430072, China; 2 Institute for Social Science Research, The University of Queensland, 80 Meiers Road, Indooroopilly 4068, QLD, Australia; 3 Department of Sociology, Wuhan University, 299 Bayi Road, Wuhan 430072, China; 4 College of Economics and Management, Southwest University, 2 Tiansheng Road, Chongqing 400715, China

**Keywords:** corporate social responsibility, collectivism, individualism, organizational pride, perceived organizational support, work engagement

## Abstract

We investigate the different mechanisms concerning how employees’ perceptions of external and internal corporate social responsibility (CSR) serve to influence employees’ work engagement. By combining social exchange theory and social identity theory, we implement and examine an integrated moderated mediation framework in which employees’ value orientations (e.g., collectivism or individualism) impact the mediating mechanism between their perceived external and internal CSR, organizational pride and perceived organizational support (POS), and work engagement. This work fills a research gap to examine the indirect relationship between employees’ perceptions of external and internal CSR and work engagement. Using two periods of survey data from 250 working employees in China, we find that employees’ perceptions of external CSR positively influence work engagement via organizational pride. The value of collectivism strengthens the direct effect of employees’ perceptions of external CSR on work engagement, and the indirect effect of employees’ perceptions of external CSR on work engagement via organizational pride. Moreover, employees’ perceptions of internal CSR positively influence work engagement via POS. The value of individualism strengthens the direct effect of employees’ perceptions of internal CSR on work engagement, and the indirect effect of employees’ perceptions of internal CSR on work engagement via POS. The results contribute to both theory and practice.

## 1. Introduction

The importance of work engagement to employee well-being and organizational productivity has been investigated in detail [1,2,3,4]. Work engagement refers to a positive and fulfilling work-related mental state, and is characterized as full of vigor, dedication, and absorption [5,6]. Extant studies imply a positive link between employees’ perceptions of corporate social responsibility (CSR) and their work engagement [7,8,9,10,11]. However, the most relevant studies have mainly tested the direct relationship between CSR and work engagement, without considering the possible mechanism under which the effect occurs. Ignoring this possible mechanism may limit the practical implications of the study, and impair further investigation of this research. In order to address these issues, we attempt to reveal the multiple mediation mechanisms and possible boundary conditions through which CSR influences work engagement.

Firstly, most studies pertinent to CSR and work engagement set CSR as a unitary structure [7,9,12]. However, existing studies suggest that employees may distinguish CSR according to different stakeholders, instead of treating it as a unidimensional construct [13]. If employees differentiate CSR based on the various target groups, then this means that employees might be affected through different processes and mechanisms by diverse CSR actions. Consequently, in accordance with the classification method used in macro CSR research, we divide CSR into external and internal CSR, and then conduct research [14,15].

Secondly, prior studies have explained the direct linkage between CSR and work engagement mostly through social identity theory [8,10,11]. However, there may be other theoretical explanations for the relationship between micro-CSR and employee work engagement. Some scholars believe that multiple mechanisms may be more accountable for the relationship between CSR and work engagement [8]. Therefore, by combining social identity theory and social exchange theory, we construct a multi-dimensional model to explore the different impact of internal and external CSR on employee work engagement. Specifically, based on social identity theory, we predict that external CSR will affect the employees’ work engagement via organizational pride, and we hypothesize that internal CSR will affect employees’ work engagement via perceived organizational support (POS) according to social exchange theory.

Thirdly, if internal and external CSR can affect employees’ work engagement through social identity and social exchange mechanisms, the boundary conditions of these processes may also vary. We argue that employees’ value orientations (e.g., collectivism and individualism) are potential boundary conditions through which CSR influences employees’ work engagement. Existing studies have explored the multiple boundary conditions through which CSR influences employees’ organizational identification, based on the mechanism of social identity [14,16]. However, there is still a lack of research exploring possible boundary conditions under other mechanisms (e.g., social exchange mechanisms). Addressing the possible boundary conditions under the social exchange mechanism is also worthwhile, since it may help managers develop better CSR strategies [17,18]. Based on social identity theory, we predict that when employees with higher collectivism orientation, the indirect effect of external CSR on work engagement via organizational pride will be strengthened. While basing on social exchange theory, we predict that when employees with higher individualism orientation, the indirect effect of internal CSR on work engagement via POS will be strengthened as well.

Present research analysis implies that collectivism moderates the indirect relationship between external CSR and work engagement via organizational pride. Individualism impacts the indirect effect of internal CSR on work engagement. POS mediates the relationship between internal CSR and work engagement. 

The study contributes to both theory and practice. This study refines the literature by expounding on how perceived CSR (both internal and external) influences employees’ work engagement. In so doing, this research shows how the underlying mechanisms of function-organizational pride, and perceived organizational support, connect components of CSR and employees’ work engagement. Furthermore, investigating the mediating mechanisms could provide momentum for firms’ CSR initiatives. This implies that in order to devise effective CSR strategies, managers must take into account the differential impact of internal and external CSR on employees. Besides, in suggesting that the mediating effect relies on employees’ value orientation (collectivism or individualism), this study shows how value orientation acts as a boundary condition of the underlying mechanism between perceived CSR and staff work engagement. 

The remainder of this study is structured as follows. First, insights from the social identity theory and social exchange theory are integrated to develop relevant hypotheses on the effect of employees’ perceptions of external and internal CSR on work engagement. Second, the methodology is outlined and the empirical findings are presented. Finally, the study’s contributions and suggestions for future research are discussed.

## 2. Theory and Hypotheses

### 2.1. Internal CSR and External CSR

Due to the continuous development of the economy and society, the definitions and meanings of CSR are constantly changing. In order to avoid being confused by the multiple concepts of CSR, this study adopts the definition of Waldman et al. CSR refers to the behavior of corporations to protect or promote social welfare beyond the direct interests of corporations and their stakeholders as stipulated by law [19]. The majority of scholars believe that CSR is a multi-dimensional concept related to different corporate stakeholders, including communities, employees, consumers, and the environment [13,14,20]. Some of these CSR beneficiaries belong to the corporation’s internal stakeholders, while others belong to the corporation’s external stakeholders. Therefore, this study divides the employees’ perceived CSR into internal CSR and external CSR. This classification method is consistent with macro CSR researchers and “Green Paper: Promoting a European framework for Corporate Social Responsibility” from Commission of European Communities [21,22].

External CSR refers to social responsibility actions targeted at local community, natural environment, and consumers [14,23]. Among them, CSR related to the community includes charitable donations in support of humanitarian causes, community development investment, and cooperation with non-government organizations [20,24]. CSR related to the environment includes investments that are related to environmental protection, such as reducing pollution, initiatives for environmental protection, and practices that focus on sustainable development for future generations [13,20]. CSR committed to the consumer includes providing quality goods or services, care commitments to customers, and protecting the interests of consumers beyond the requirements of the law [13,14].

Internal CSR refers to the actions that corporations choose to take to satisfy the expectations of employees, actively fulfill and improve organizational fairness regarding employees (such as improving the happiness and satisfaction of employees’ health), and ensure work safety and the growth and development of employees [25,26,27]. By providing welfare services for employees, internal CSR is closely related to psychological and physiological wellbeing, and the core idea of CSR is to benefit employees instead of pursuing corporate interest [14,27].

### 2.2. External CSR and Work Engagement: Based on Social Identity Perspective

Work engagement refers to employees’ cognition, emotion, and behavior at work [6,28]. It is an emotional and psychological expression of vigor, dedication, and absorption [1]. In many studies, employee engagement has been regarded as an effective indicator to reflect the quality of employees’ work (such as performance, satisfaction) and to affect employees’ career development [29]. Organizational pride has been defined as “the extent to which individuals experience a sense of pleasure and self-respect arising from their organizational membership” [30].

Social identity theory suggests that the identification of an individual with an organization is often acquired through association and comparison with other people or organizations [31]. The purpose of an individual’s identification with a specific organization is to achieve self-worth improvement [32]. Employees can use the reputation or social status of the organization to evaluate their expectations of self-improvement [33]. Therefore, employees are more inclined to identify with organizations that have a good image to meet self-improvement needs and enhance self-value. CSR for external stakeholders such as the community, environment, and consumers is not only a form of moral behavior beyond their own economic interests but also a reflection of a healthy image and reputation [34]. By perceiving external CSR, employees can predict the improvement of their self-value. Employees can then acquire a high level of organizational pride through comparison and self-feedback with CSR of other organizations [30]. In turn, organizational pride can fulfill social identity needs, thus retaining employee engagement [35]. 

Existing studies have confirmed that CSR can influence employees’ attitudes and behaviors through improving their organizational pride. For example, Jones found that CSR affects employees’ attachment to and pride in their organization, and thus affects their work-related attitudes and behavior [30]. Rupp and others found that the work engagement of employees increases with the implementation of corporate social responsibility, because employees become prouder of the enterprise [7]. In addition, scholars have also indicated that when an organization has a healthy reputation outside the workplace, employees not only have a high level of self-evaluation with regard to themselves but their sense of pride and belonging to the organization is also enhanced [35]. This contributes to high work engagement [9]. Thus, we propose the following hypothesis:

**Hypothesis 1** **(H1):** Organizational pride mediates the effect of employees’ perceptions of external CSR on work engagement.

### 2.3. Internal CSR and Work Engagement: Based on Social Exchange Perspective

CSR has an instinctive need to promote the social exchange process between organizations and employees [36,37]. Therefore, corporate social responsibility may affect the attitudes and behaviors of employees through social exchange mechanisms [22,38]. A basic principle of social exchange theory is the assumption of reciprocity: if a person provides something of value, the recipient should return in kind [39]. In the case of social exchange, one party voluntarily provides benefits to the other party and evokes an obligation by providing some benefits in return [38,40]. The principle of reciprocity is particularly applicable to internal corporate social responsibility, since it leads to some companies taking action beyond strategic human resource management. This will be used to support employee welfare and sustainability [23,30,41]. Hence, employees may feel obligated to repay these voluntary investments.

Internal CSR is based on voluntary behavior of a corporation, and does not require employees to return this in kind. [13,42]. Corporate actions of helping employees, such as providing fair treatment, organizing good working environment training, and career development opportunities, can provide employees with a strong sense of organizational support [43]. Based on the principle of reciprocity, employees will exert more engagement at work to reciprocate to corporations. Thus, employees will be psychologically, cognitively, and behaviorally engaged in the workplace [8]. Prior research has also shown that, based on the principle of reciprocity and exchange, employees work harder to reward corporations (such as through enhancing trust, commitment, and organizational citizenship behavior) when employees perceive that corporations have gratified their relevant needs, [23,30]. Based on the discussion above, we propose:

**Hypothesis 2** **(H2):** POS mediates the effect of employees’ perceptions of internal CSR on work engagement.

### 2.4. Moderating Role of Employee Collectivism and Individualism

Collectivism and individualism are two different types of belief and value systems in organizational culture. They are useful for explaining cultural differences, and they have an important influence on individuals’ levels of motivation, attitudes, and behavior [44,45]. Collectivism, concentrated on group connection and mutual obligations among individuals, is a culture and value system that focuses on collective goals. Furthermore, it hopes to ensure harmony with others by keeping in line with the other members of the organization [46]. Collectivism emphasizes group identity, interpersonal belonging, and emotional support. It focuses on the overall results of individuals and groups [47]. Individualism is centered on the expression of individual independence and internal characteristics, and is a culture and value system that focuses on individual goals, uniqueness, and self-development [46]. Individualism emphasizes the supremacy of individual interests, pays more attention to the actualization of individual goals and interests, and believes that work is an important way to actualize individual value and individual development [47].

Collectivists prioritize group interests rather than individual interests. They focus on socially oriented goals [47]. Since the social exchange mechanism is more relevant to the interests of individuals [38], and the social identity mechanism is more related to group identity [32], we thus predict that individualism is more likely to affect the social exchange mechanism between employees and organizations. On the other hand, collectivism has a higher possibility to influence the social identity mechanism between employees and organizations. Specifically, employees with a higher collectivism tendency regard organizations and groups as a source of identity, and attach great importance to society-oriented goals and the welfare of their organization [45,48]. Hence, those employees that value collectivism have a higher perception of organizational pride when their corporations adopt more social responsibility for external stakeholders such as the community and environment [30]. In addition, scholars have also found that external CSR, especially that that focuses on social welfare, functions as a special source to increase the organizational identity of employees’ that are collectivists, and thus impact their work performance [14]. Based on the assumption that the positive impact of employees’ perception of external CSR on organizational pride is stronger when employees operate at a high level of collectivism, and that employees’ organizational pride is positively related to work engagement, it is logical to speculate that the positive indirect effect of employees’ perception of external CSR on work engagement via organizational pride will be stronger when employees have a higher collectivist tendency. As a result, we propose the following hypotheses:

**Hypothesis 3a** **(H3a):** Collectivism moderates the relationship between employees’ perceptions of external CSR and organizational pride. Specifically, the relationship between external CSR and organizational pride will be reinforced when individuals hold a higher level of collectivism.

**Hypothesis 3b** **(H3b):** Collectivism moderates the indirect relationship between employees’ perceptions of external CSR and work engagement by means of organizational pride. Thus, the indirect effect will be stronger when employees have a higher collectivist tendency.

Individualists pay more attention to their own interests and material needs [49]. They do not have a strong sense of community belonging, and do not care about social image and social status. They pay more attention to the realization of personal interests and goals [50]. Therefore, they fulfill individual internal CSR by helping, supporting, and respecting their vital interests and development. Internal CSR is more sensitive to employees with a higher level of individualism [14]. Thus, employees will be more dedicated and involved in their work. Some studies have verified that the relationship between employees’ perceptions of internal CSR and their work engagement is enhanced among employees that place a greater emphasis on individualism [7]. Based on the notion that the positive relationship between employees’ perception of internal CSR and POS is stronger when employees have a higher level of individualism, and that employees’ POS is positively related to work engagement, it is logical to posit that the positive indirect effect of employees’ perception of internal CSR on work engagement via POS will be stronger when the individualism orientation of employees is high. From the above arguments, we hypothesize the following:

**Hypothesis 4a** **(H4a):** Individualism moderates the relationship between employees’ perceptions of internal CSR and POS, such that the relationship will be strengthened when employees hold a higher level of individualism.

**Hypothesis 4b** **(H4b):** Individualism moderates the indirect effect of employees’ perception of internal CSR on work engagement via POS. Specifically, the indirect effect will be improved under a high level of individualism.

**Figure 1:**
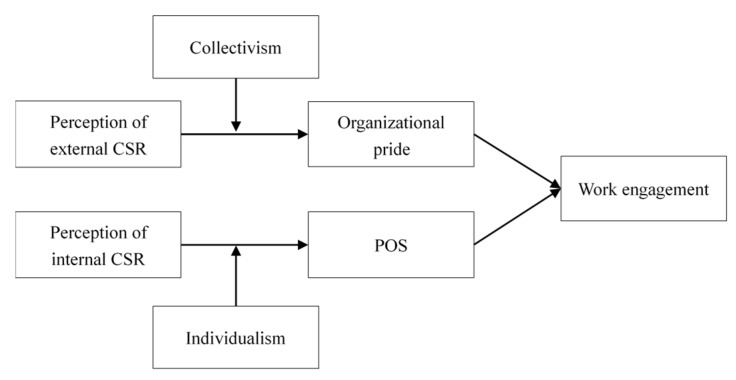
The conceptual model.

The four specific hypotheses are shown in Figure 1. 

## 3. Methods 

### 3.1. Procedure and Sample

An appropriate sample is the benchmark for making this research successful. In order to decrease common methods of variance and better investigate the causal relationship between each variable, a cross-sectional survey was conducted to collect data basing on longitudinal research design. The participants in this study were employed by a professional survey company. They used unique collaborative recommendation models to improve efficiency and quality of data collection. The survey company randomly selected 1000 participants from our survey out of its database, which included approximately 7,800,000 panelists. This reduced the possibility of biased sampling. An English-language version of the questionnaire was first prepared, and then, after going through a double translation process, it was translated into Chinese. The numbers of screening questions were used to ensure only qualified respondents participated in the survey. For example, since our questionnaire related to the firm’s CSR activities, some samples were excluded in relation to the following question in the questionnaire: “Are there CSR behaviors in your company?”. 

The whole investigation was split into two periods. In the first period, a questionnaire measured the perceived corporate social responsibility (CSR), individualism value, collectivism value, and some control variables (e.g., gender, age, marriage, education, firm size, and industry type). This was sent to participants via email. Finally, 277 valid questionnaires were returned. Half month later, a second period survey was executed. Questionnaires were sent to the 277 participants again, assessing individual’s work engagement, POS, and organizational pride. Eventually, 250 valid questionnaires were collected. We received 250 questionnaires from 221 firms covering 22 Provinces in China. For the sample as a whole, the industry included manufacturing (62.0%), financial (17.2%), retailing (10.4%), and others (10.4%). The firms with assets of more than 50 million formed 71.60% of the sample. Among all 250 employees, 50.40% were female and 49.60% were male. The proportion of those aged below 25 was 16.4%, 25–35 was 30.8%, 35–45 was 22.8%, 45–55 was 24.0%, and above 55 was 6.0%. Unmarried employees constituted 5.20%, whereas married employees constituted 94.8%. The education level included high school (12.0%), undergraduate degree (73.2%), and master’s or above (24.8%).

### 3.2. Measures

#### 3.2.1. Perceived Corporate Social Responsibility (CSR) 

Employees’ perceptions of their corporate social responsibility were assessed by perceived internal and external CSR scales. These were revised by Farooq and his colleagues in 2017 [14]. All items were measured on a seven-point Likert scale, ranging from totally disagree (1) to totally agree (7). Perceived external CSR was assessed through three dimensions: environment-targeted CSR (Cronbach’α = 0.927), community-targeted CSR (Cronbach’α = 0.923), and consumer-targeted CSR (Cronbach’α = 0.926). Each dimension consisted of four items, three items, and three items, respectively. Confirmatory factor analysis (CFA) showed that this scale has a good model fit, Ratio of chi-squared to degrees of freedom(χ^2^/df) = 2.469, Root Mean Square Error of Approximation (RMSEA) = 0.077, Goodness of Fit Index (GFI) = 0.984, and Tucker-Lewis Index(TLI) = 0.975. Perceived internal CSR refers to employee-targeted CSR. This was measured across six items in one dimension (Cronbach’α = 0.925). The alpha coefficient of the total sixteen-item scale was 0.922 (Table 1).

#### 3.2.2. Work Engagement 

We relied on Schaufeli’s short version scale [5], including a total of nine items. The instrument was split into three dimensions (vigor, dedication, and absorption) with three items, respectively. All the items were rated on a seven-point Likert scale, ranging from totally disagree (1) to totally agree (7). The entire alpha coefficient of this scale was 0.955. Confirmatory factor analysis (CFA) showed that this scale had a good model fit (χ^2^/df = 2.003, RMSEA = 0.063, GFI = 0.992, and TLI = 0.984). The Cronbach’α were 0.919, 0.926, and 0.925 for vigor subscale, dedication subscale, and absorption subscale, respectively. This indicates that each dimension of this scale had convincing reliability.

#### 3.2.3. Perceived Organizational Support (POS) 

The POS is a four-item, one-factor designed scale from Rosso et al. [51]. All the items were measured on a seven-point Likert scale, ranging from 1 (totally disagree) to 7 (totally agree). The alpha coefficient was 0.883 in the present study.

#### 3.2.4. Organizational Pride 

We measured organizational pride with four items adapted from Jones [30], with an alpha coefficient of 0.891. All the items were measured on a seven-point Likert scale, ranging from 1 (totally disagree) to 7 (totally agree).

#### 3.2.5. Collectivism

We adopted Triandis’ scales to evaluate collectivism [47]. Collectivism comprises eight items. The Cronbach’α of the entire scale was 0.973. All the items were measured on a seven-point Likert scale, ranging from 1 (totally disagree) to 7 (totally agree).

#### 3.2.6. Individualism

We adopted scales to evaluate individualism and collectivism from Triandis [47]. Individualism consisted of eight items, and the Cronbach’α of the entire scale was 0.977. All items were measured on a seven-point Likert scale, ranging from 1 (totally disagree) to 7 (totally agree).

#### 3.2.7. Control Variables

To isolate potential confounding effects, in addition to the demographic information on the employees, we also controlled for firm size and industry type. This was because previous research had shown that these factors were positively associated with the consequences of CSR [12,52,53]. Firm assets were used to measure firm size [54,55].

### 3.3. Data Analysis

The data was analyzed using SPSS 21 (IBM, Armonk, NY, USA) and Mplus 6 (Muthen & Muthen, Los Angeles, CA, USA). Confirmatory factor analysis and reliability analysis were conducted to test the appropriateness of used scales. We followed Hair et al. (2010) in measuring the discriminant and convergent validities of all the scales. Construct Reliability (CR) > 0.7 and Average Variance Extracted (AVE) > 0.5 were adopted to establish reliability and convergent validity, respectively (see Table 1). Multiple fit indices were used to evaluate the goodness-of-fit of each scale: the χ^2^ and Bollen–Stine bootstrap p value of 0.05 or greater, indicating an appropriate fit; a comparative fit index (CFI) with value ≥0.9, indicating an appropriate fit [56]; and a Root Mean Square Error of Approximation (RMSEA) with values ≤0.08, indicating an acceptable fit of the model to the data [57,58]. 

Mediating and moderating analysis were combined to assess the relationship between CSR, work engagement, POS, organizational pride, collectivism, and individualism.

### 3.4. Ethical Approval

This research belongs to the social science domain, and the scales adopted in this research do not require permission to be used. This research received ethical approval from Occupational Mental Health Promotion Committee of Chinese Association for Mental Health. All subjects gave their informed consent for inclusion before they participated in the survey. The data from this research has been strictly maintained to ensure their confidentiality, and will not be disclosed to any third party. No personal information has been mentioned in the reporting and publication.

## 4. Results 

### 4.1. Common Method Variance

Since the data for this research were collected on self-reported measures, they may have caused common method variance. In consequence, we conducted two methods to test the impact of common method bias. Firstly, we adopted Harman’s single-factor analysis using exploratory factor analysis [59]. Here, we combined all the variables into one factor. The results showed that the single factor explained only 25.733% of the variance. Secondly, we conducted Harman’s factor analysis again, but this time we extracted factors according to their eigenvalues, instead of fixing them to one factor. The result indicated that the items yielded seven factors. This explained 74.365% of the total variance. Among these factors, the first factor was accountable for 26.371% of the variance. This is far less than the 50% standard proposed by Hair et al. [60].

### 4.2. Descriptive Analysis

Table 2 is a descriptive analysis and generates the means, standard deviations, and correlations among the variables used in this research, providing readers with a basic insight into the present research.

### 4.3. Hypotheses Testing

Hypothesis 1 and Hypothesis 2 explored the mediating effect of POS and organizational pride on CSR (ECSR and ICSR) and work engagement. The whole mediating model showed a good model fit (χ^2^/DF = 1.902, RMSEA = 0.052, CFI = 0.940, and TLI = 0.934). For Hypothesis 1, we found that external CSR wields a positive influence on organizational pride (estimation = 0.539, SE = 0.093, *p* < 0.001) and work engagement (estimate = 0.146, SE = 0.069, *p* < 0.05). Furthermore, organizational pride also exerts a positive effect on work engagement (estimate = 0.237, SE = 0.070, *p* < 0.01), and external CSR has a significantly positive indirect influence on work engagement through organizational pride (estimate = 0.128, SE = 0.044, and *p* < 0.01). The results indicate that organizational pride partially mediates the relationship between external CSR and work engagement. This supports Hypothesis 1 (see Table 3). For Hypothesis 2, internal CSR had a significant effect on POS (estimate = 0.316, SE = 0.077, *p* < 0.001) and work engagement (estimate = 0.299, SE = 0.063, *p* < 0.001). POS also had a positive influence on work engagement (estimate = 0.216, SE = 0.059, and *p* < 0.001), and internal CSR had a remarkable and positive influence on work engagement through POS (estimate = 0.068, SE = 0.026, and *p* < 0.01). The results suggest that POS partially mediates internal CSR and work engagement, which is demonstrated in Hypothesis 2 (see Table 3).

In order to test Hypothesis 3 with Hypothesis 4, we adopted the most recently used PROCESS macro for SPSS with 5000 bootstrap samples. This was pertinent for calculating the interaction effects. As we hypothesized that the moderator only affects the first-stage relationships, we used Model 7 (i.e. a moderated mediation model). This will be convincing if two factors are met. First, if the interaction variables [e.g., external CSR multiplied by collectivism (Hypothesis 3a) and internal CSR multiplied by individualism (Hypothesis 4a)] are significantly related to the mediator (e.g., organizational pride and POS). Second, if the conditional indirect effect of external CSR on work engagement through organizational pride differs in strength across low, mean, and high levels of collectivism (Hypothesis 3b); and the conditional indirect effect of internal CSR on work engagement through POS differs in strength across low, mean, and high levels of individualism (Hypothesis 4b) (see Table 4 and Table 5).

**Figure 2:**
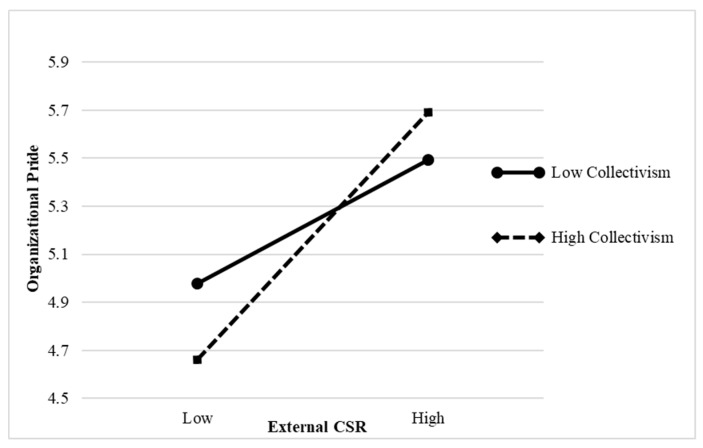
The interactive effect of external corporate social responsibility (CSR) and collectivism on organizational pride.

**Figure 3:**
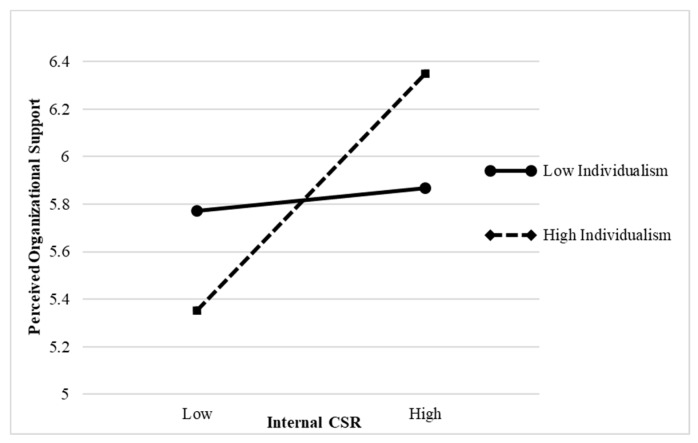
The interactive effect of internal CSR and individualism on perceived organizational support (POS).

Collectivism positively moderated the relationship between employees’ perceptions of external CSR and organizational pride (interaction effect = 0.161, *p* < 0.01); therefore, Hypothesis 3a is supported (see Table 4). This result is further confirmed in Figure 2. When collectivism is high, employees’ perceptions of external CSR are more positively related to organizational pride than when collectivism is low. Moreover, individualism significantly moderated the relationship between employees’ perceptions of internal CSR and POS (interaction effect = 0.230, *p* < 0.001); therefore, Hypothesis 4a is supported (see Table 4). Hypothesis 4a is further confirmed in Figure 3. Thus, when individualism is high, employees’ perceptions of internal CSR are more positively related to POS than when individualism is low.

The indirect effect of employees’ perceptions of external CSR on work engagement through organizational pride is enhanced when employees have a higher tendency towards collectivism (low: 0.082, mean: 0.162, and high: 0.179; CIs do not include zero), and the effect coefficients vary significantly. This result provides supportive evidence for Hypothesis 3b (see Table 5). In addition, result also reveals that the indirect effect of employees’ perceptions of internal CSR on work engagement through POS was stronger when employees expressed more individualism (low: 0.006, mean: 0.074, high: 0.167, CIs do not include zero), and when the effect coefficients varied significantly. Through this analysis, Hypothesis 4b is well demonstrated (see Table 5).

Based on the methods suggested by Li [61] for dealing with endogeneity problems, we also discuss the potential endogeneity issue. Li revealed that the addition of meaningful control variables appears to work, even without a valid instrumental variable. We compare the results of our models (i.e., mediating effect model, moderating effect model, and moderated mediation model) before and after the addition of control variables. We find the results are same. Hence, the endogeneity problem is minimal in our research.

## 5. Discussion 

All four hypotheses have been verified. Organizational pride mediates the relationship between employees’ perceptions of external CSR and work engagement, and collectivism moderates the direct effect between employees’ perceptions of external CSR and organizational pride. When employees hold a high-level collectivist attitude, the indirect impact on employees’ perceptions of external CSR expressed to work engagement will be strengthened. Moreover, employees’ perceptions of internal CSR influences work engagement through POS. Additionally, individualism moderates the direct effect employees’ perceptions of internal CSR has on POS. The higher employees’ individualism is, the stronger indirect influence employees’ perceptions of internal CSR exerts on work engagement.

### 5.1. Theoretical Contributions

The theoretical contributions of this research are as follows. First, we built a new research framework by dividing employees’ perceptions of CSR into internal CSR and external CSR according to the different CSR stakeholders. This responded to the recent call for macro CSR researchers [14,21,22]. We then used this framework to explore the different effect mechanisms of employees’ perceptions of internal and external CSR on employees’ work engagement.

Second, we developed a new theoretical interpretation mechanism for investigating the relationship between employees’ perceptions of CSR and employees’ work engagement. Major studies on the relationship between employees’ perceptions of CSR and employees’ work engagement are based on social identity theory, but we expanded on previous studies [14,16] in our work by combining social identity theory and social exchange theory so that it could provide a solid answer to this question.

Third, this study clarifies the boundary conditions of employees’ perceptions of CSR influencing employees’ work engagement under different mechanisms. Besides, this study responds to the call for exploring CSR from the perspective of value orientation [7] by considering the moderating effects of an individual’s value orientations (i.e., collectivism and individualism). Specifically, if employees value collectivism, their perception of external CSR will lead to higher work engagement through gaining more organizational pride. If employees are individualists, their perception of internal CSR will promote work engagement through the POS.

### 5.2. Managerial Implications

Our findings have implications for managers when they develop and implement CSR strategies. Firstly, internal and external CSR strategies are equally essential to promoting positive performances. This is because employees’ attitudes and behaviors are valuable, rare, inimitable, and non-substitutable [62,63]. Thus, by motivating employees’ work engagement, both internal and external CSR strategies can help organizations maintain effective labor resources, create competitive advantages, and effectively enforce organizational business strategies. Thus, overall organizational performance can be improved. Secondly, our findings suggest that the benefits of CSR activities are not limited to gaining external reputation and support from external stakeholders, but also help to change the attitudes of internal employees. Both internal and external CSR strategies effectively enhance employees’ work engagement. Hence, when making CSR strategic decisions, managers should not only fulfill the external CSR to increase the employees’ work engagement through stimulating the organizational pride of employees but also satisfy employees’ personal needs. This will improve their work engagement through POS. Thirdly, the findings suggest that the impact of CSR on employees’ work engagement varies from person to person. Thus, our results can help managers develop more effective and targeted CSR strategies. Managers should keep these individual differences in mind when assessing the role of CSR. For example, our findings show that community-targeted CSR practices have a stronger predictive effect on employees who are collectivists. Therefore, when developing CSR strategies, it is wise for managers to consider organizational cultural values and employees’ characteristics. Managers should create the CSR strategy based on the reality of their employees, and strive to maximize the positive impact on employees.

### 5.3. Limitations and Future Research

We combined social identity theory and social exchange theory and verify the relationship between employees’ perceptions of internal and external CSR and work engagement. Although we obtained some meaningful research conclusions, there are still some limitations and areas for further study. This is because many uncontrolled factors remain. First, we explored the impact of employees’ perceptions of internal and external CSR on work engagement. However, we ignored employees’ other attitudes and behaviors, such as the effect of employees’ perceptions of internal and external CSR on employees’ negative emotions and behaviors. Future research could simultaneously explore the influence of CSR on a variety of employees’ attitudes and behaviors (e.g., negative and positive attitudes and behaviors), to better clarify the extent and impact of the employees’ perceptions of internal and external CSR on different work attitudes and behaviors. In addition, we only focused on the employee level to explore the impact of employees’ perceptions of internal and external CSR on work engagement, while ignoring leader-level and firm-level factors, such as CEO characteristics [52,55,64]. Future research about CSR could explore the multilevel effect, such as simultaneously considering the characteristics of employees, leaders, firms, and industries. For example, how does CSR affect companies operations in different sectors and their workers? Moreover, we only used one data source, and this may have resulted in the risk of common method bias. In order to deal with these issues, we incorporated some of the procedural remedies proposed by Podsakoff et al. [59] to reduce common method biases. Test results have shown that common method bias does not seem to affect our findings. Nevertheless, we suggest that future research combine experimental and longitudinal research methods to obtain stronger causal inferences. Finally, this study is based on a Chinese sample. Many of the hypotheses proposed in the study are supported; however, we cannot promote our findings to other countries based solely on this research. Hence, we recommend further research to test this framework in different national contexts.

## 6. Conclusions

To date, extant empirical studies regarding CSR research have attested that perceived that CSR can be an incentive for employees’ workplace outcomes, such as employees’ work engagement and commitment to organizations. Although these findings do support the relevance of CSR to work engagement, researchers mostly examine the direct relationship, the way how CSR functions and its influential mechanisms, especially the indirect pathways are still unknown. In order to address this limitation, present research builds an integrated moderated mediation framework to investigate the mechanism of how employees’ perceptions of external and internal CSR facilitate their work engagement. Specifically, this research combines social exchange and social identity theory to form the basic framework for the entire model. It then tests the moderated mediation model in which employees’ value orientations (e.g., collectivism or individualism) moderate the mediating mechanism between their perceived CSR, organizational pride, and perceived organizational support, together with work engagement. Statistical analysis indicates that employees’ perceptions of external CSR positively influenced work engagement via organizational pride. Collectivist value strengthened the direct effect of employees’ perceptions of external CSR on work engagement, and the indirect effect of employees’ perceptions of external CSR on work engagement through organizational pride. Moreover, employees’ perceptions of internal CSR positively influenced work engagement via POS. Individualist value strengthened the direct effect of employees’ perceptions of internal CSR on work engagement, and the indirect effect of employees’ perceptions of internal CSR on work engagement via POS. 

The results demonstrate the conceptual model at large. They contribute both theoretically and practically to the ongoing debates. According to the present literature, there is a dearth of studies pertinent to how internal and external CSR impacts employees’ attitudes, value orientations, and behaviors—especially the indirect effects CSR functions. These results extend the sparse knowledge on the indirect relationship between CSR and work engagement, and fill the gap of how CSR functions. This contribution is vital in the sense that it allows us to reveal the missing linchpins between CSR and work engagement. Besides, this research can also provide some insights for managers to formulate and implement CSR strategies. This research suggests that though internal and external CSR both contribute to employees’ work engagement, their influential mechanisms differ, so managers should distinguish these external and internal CSR clearly. In addition, findings also demonstrate that managers can institute their CSR strategies according to employees’ value orientation as a way to profoundly motivate them.

## Figures and Tables

**Table 1:** Reliability and validity.

	Factor Loading	CR	AVE
**External CSR**		0.962	0.715
ECSR1	0.850		
ECSR2	0.786		
ECSR3	0.838		
ECSR4	0.852		
ECSR5	0.864		
ECSR6	0.851		
ECSR7	0.837		
ECSR8	0.855		
ECSR9	0.867		
ECSR10	0.855		
**Internal CSR**		0.926	0.675
ICSR1	0.830		
ICSR2	0.845		
ICSR3	0.773		
ICSR4	0.846		
ICSR5	0.838		
ICSR6	0.794		
**POS**		0.884	0.657
POS1	0.813		
POS2	0.846		
POS3	0.823		
POS4	0.758		
**Organizational pride**		0.891	0.672
OP1	0.815		
OP2	0.835		
OP3	0.844		
OP4	0.783		
**Work engagement**		0.955	0.703
WE1	0.844		
WE2	0.830		
WE3	0.830		
WE4	0.833		
WE5	0.847		
WE6	0.835		
WE7	0.847		
WE8	0.834		
WE9	0.846		
**Individualism**		0.977	0.842
IND1	0.931		
IND2	0.928		
IND3	0.907		
IND4	0.906		
IND5	0.896		
IND6	0.911		
IND7	0.921		
IND8	0.938		
**Collectivism**		0.973	0.821
COL1	0.923		
COL2	0.884		
COL3	0.909		
COL4	0.887		
COL5	0.894		
COL6	0.918		
COL7	0.915		
COL8	0.917		

Note: External CSR: external corporate social responsibility; Internal CSR: internal corporate social responsibility; POS: perceived organizational support; CR: Construct Reliability; AVE: Average Variance Extracted.

**Table 2:** Descriptive statistics, mean, standard deviation, and correlations.

Variables	Mean	SD	1	2	3	4	5	6	7
1.ECSR	5.248	0.767	1						
2.ICSR	5.143	0.952	0.250 **	1					
3.EN	5.275	0.790	0.411 **	0.493 **	1				
4.OP	5.266	0.851	0.482 **	0.242 **	0.433 **	1			
5.POS	5.118	0.933	0.338 **	0.289 **	0.461 **	0.353 **	1		
6.COL	5.067	1.049	−0.014	−0.048	−0.081	−0.055	−0.028	1	
7.IND	2.682	1.034	−0.027	−0.045	−0.094	−0.037	0.010	0.185 **	1

Notes: *N* = 250; * *p* < 0.05; and ** *p* < 0.01. ECSR: external corporate social responsibility; ICSR: internal corporate social responsibility; EN: work engagement; OP: organizational pride; POS: perceived organizational support; IND: collectivism; and COL: individualism; SD: Standard Deviation.

**Table 3:** Mediation analysis results.

Indirect and Direct Effect	Estimate	S.E.	BC 95% CI	
			Lower	Upper
**Indirect effect**				
ECSR→OP→EN	0.128 **	0.044	0.055	0.231
ICSR→POS→EN	0.068 **	0.026	0.029	0.134
**Direct effect**				
ECSR→EN	0.146 *	0.069	0.018	0.292
ICSR→EN	0.299 ***	0.063	0.189	0.436
ECSR→OP	0.539 ***	0.093	0.367	0.729
ICSR→POS	0.316 ***	0.077	0.169	0.532
OP→EN	0.237 **	0.070	0.106	0.384
POS→EN	0.216 ***	0.059	0.113	0.348

Notes: BC: biased corrected (5000 bootstrapping sample). Control variables (gender, age, education, marriage, firm size, and industry type) are included in the model. * *p* < 0.05, ** *p* < 0.01, and *** *p* < 0.001. ECSR: external corporate social responsibility; and ICSR: internal corporate social responsibility; SE: standard error.

**Table 4:** Moderating analysis results.

	Estimate	S.E.	BC 95% CI		Estimate	S.E.	BC 95% CI	
			Lower	Upper			Lower	Upper
ECSR	−0.313	0.325	−0.952	0.326				
COL	−0.873 **	0.324	−1.512	-0.234				
ECSR * COL	0.161 **	0.062	0.039	0.282				
ICSR					−0.326	0.166	−0.652	0.002
IND					−1.167 ***	0.304	−1.767	−0.568
ICSR * IND					0.230 ***	0.058	0.115	0.345

Notes: BC: Biased Corrected (5000 bootstrapping sample). Control variables (gender, age, education, marriage, firm size, and industry type) are included in the model. * *p* < 0.05, ** *p* < 0.01, and *** *p* < 0.001. ECSR: external corporate social responsibility; ICSR: internal corporate social responsibility; EN: work engagement; op: organizational pride; POS: perceived organizational support; COL: collectivism; IND: individualism.

**Table 5:** Moderated mediation results.

	ECSR→OP→EN				ICSR→POS→EN			
	Estimate	S.E.	BC 95% CI		Estimate	S.E.	BC 95% CI	
			Lower	Upper			Lower	Upper
COL low	**0.082**	0.035	0.027	0.162				
COL mean	**0.162**	0.047	0.082	0.261				
COL high	**0.179**	0.053	0.087	0.291				
Index	**0.046**	0.023	0.003	0.095				
IND low					**0.006**	0.028	-0.048	0.062
IND mean					**0.074**	0.023	0.033	0.122
IND high					**0.167**	0.045	0.086	0.261
Index					**0.068**	0.023	0.026	0.117

Notes: BC: biased corrected (5000 bootstrapping sample). Control variables (gender, age, education, marriage, firm size, and industry type) are included in the model. Estimates with CIs that do not include zero are statistically significant and bolded.
